# Reciprocal regulation of γ-globin expression by exo-miRNAs: Relevance to γ-globin silencing in β-thalassemia major

**DOI:** 10.1038/s41598-017-00150-7

**Published:** 2017-03-16

**Authors:** Kuo-Ting Sun, Yu-Nan Huang, Kalaiselvi Palanisamy, Shih-Sheng Chang, I-Kuan Wang, Kang-Hsi Wu, Ping Chen, Ching-Tien Peng, Chi-Yuan Li

**Affiliations:** 1 Graduate Institute of Clinical Medical Science, China Medical University, Taichung, 40402 Taiwan; 2 Department of Pediatric Dentistry, China Medical University Hospital, Taichung, 40402 Taiwan; 3 School of Dentistry, China Medical University, Taichung, 40402 Taiwan; 4 Department of Life Sciences, National Chung-Hsing University, Taichung, 40402 Taiwan; 5 Department of Hematology-oncology, Children’s Hospital of China Medical University, Taichung, 40402 Taiwan; 6 Division of Cardiology, Department of Medicine, China Medical University Hospital, Taichung, 40402 Taiwan; 7 Division of Nephrology, Department of Medicine, China Medical University Hospital, Taichung, 40402 Taiwan; 8 Thalassemia Research Institute, The First Affiliated Hospital, Guangxi Medical University, Guangxi Zhuang Autonomous Region, 530021 China; 9 Department of Anesthesiology, China Medical University Hospital, Taichung, 40402 Taiwan

## Abstract

Induction of fetal hemoglobin (HbF) is a promising strategy in the treatment of β-thalassemia major (β-TM). The present study shows that plasma exosomal miRNAs (exo-miRs) are involved in γ-globin regulation. Exosomes shuttle miRNAs and mediate cell-cell communication. MiRNAs are regulators of biological processes through post-transcriptional targeting. Compared to HD (Healthy Donor), β-TM patients showed increased levels of plasma exosomes and the majority of exosomes had cellular origin from CD34+ cells. Further, HD and β-TM exosomes showed differential miRNA expressions. Among them, deregulated miR-223-3p and miR-138-5p in β-TM exosomes and HD had specific targets for γ-globin regulator and repressor respectively. Functional studies in K562 cells showed that HD exosomes and miR-138-5p regulated γ-globin expression by targeting BCL11A. β-TM exosomes and miR-223-3p down regulated γ-globin expression through LMO2 targeting. Importantly, miR-223-3p targeting through sponge repression resulted in γ-globin activation. Further, hnRNPA1 bound to stem-loop structure of pre-miR-223 and we found that hnRNPA1 knockdown or mutagenesis at miR-223-3p stem-loop sequence resulted in less mature exo-miR-223-3p levels. Altogether, the study shows for the first time on the important clinical evidence that differentially expressed exo-miRNAs reciprocally control γ-globin expressions. Further, the hnRNPA1-exo-miR-223-LMO2 axis may be critical to γ-globin silencing in β-TM.

## Introduction

Fetal hemoglobin (HbF; α_2_γ_2_) is expressed in the fetal stage, however upon birth, its expression declines and adult hemoglobin (HbA; α_2_β_2_) predominates in adults^1^. β-thalassemia is a hereditary disorder with decreased (beta^+^) or complete absence (beta^0^) of β-globin chain synthesis, which leads to non-functional HbA production. Alpha-chain accumulation and resulting imbalance in α/β ratio causes ineffective erythropoiesis in β-thalassemia^2, 3^. LDB1 (LIM domain-binding protein 1)/GATA-1 (Gata binding protein 1)/LMO2 (LIM domain only 2) protein complex directly bind to locus control region (LCR) and regulate transcription of globin genes^4–6^. LMO2, a non-DNA binding protein acts as a bridging protein for LDB1, GATA1 and TAL1 (T-cell acute lymphocytic leukemia 1) and recruits the complex to the β-globin LCR^7, 8^. Transcriptional repressors, BCL11A and MYB silences HbF expression in adult erythroid progenitor cells^9–11^. Hemoglobin switching from γ to β-globin synthesis is regulated through silencing of γ-globin expression^1^. Since, hereditary persistence of HbF in adults with β-thalassemia shows less severity to the disease^12^, a promising approach to treat β-thalassemia could be through re-activation of HbF. Previous reports validate that BCL11A and MYB as targets for re-induction of HbF^9, 13–15^. The present study aims at better understanding on the γ-globin regulatory mechanism, which might be beneficial in development of specific therapeutic strategy.

miRNAs are short non-coding RNAs (approximately 22 nt), which binds to the complementary sequences and regulate gene expressions through translational repression or mRNA degradation^16^. miRNAs are regulators of physiological processes, however their differential expressions are linked to pathogenesis^17^. Evidence shows the critical role of miRNAs in HbF regulation. MiR-486-3p down regulates HbF expression through BCL11A repression, a potent negative regulator of HbF^14^. MiR-210 was found to be elevated during hereditary persistence of HbF^18^. Further, miR-15a and miR-16-1 increases HbF through down-regulation of MYB, an inhibitor of the HbF gene transcription^9^. We sought to identify whether differential miRNA expressions regulates γ-globin expression in β-thalassemia major.

miRNAs mediate intercellular communication through exosomes, in addition to their cellular regulatory roles. miRNAs and other bioactive molecules such as nucleic acids, mRNAs and proteins are packaged inside the exosomes and released into the body fluids^19^. Almost all the cells release exosomes both in normal and diseased states and cell-specific exosomes could be determined through their surface markers^20^. These circulatory exo-miRNAs are identified as disease biomarkers and evoke stimulus and cell type-specific responses in the target cells^21^. Importance of exosomes and exo-miRNAs in the hematological diseases are gaining importance. Few studies show increased circulatory microparticles in β-thalassemia and their involvement in hypercoagulability^22–24^. However, regulatory role of exosomes and exo-miRNAs in β-thalassemia major are unknown. In the present study, we aimed at identifying the role of exosomes and candidate plasma exo-miRNA mediated γ-globin regulation in β-thalassemia major.

## Results

### Characterization of cell-type specific exosome release in β-thalassemia major patients

**Figure 1:**
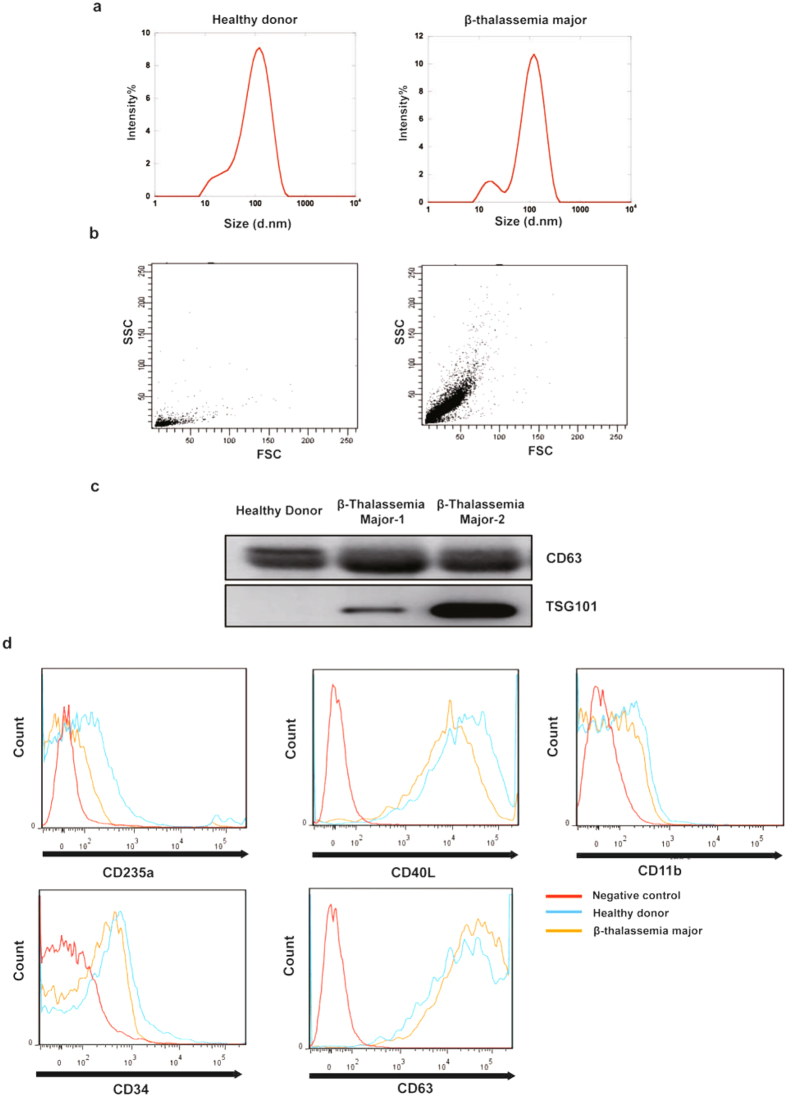
Characterization of plasma exosomes in healthy donor and β-thalassemia major patients. (**a**) Particle size distributions of exosome as measured by DLS. (**b**) Exosome counts were determined by flow cytometry and FSC-SSC data were acquired in log mode (n = 10). (**c**) Western blot for exosomal marker expression (CD63, TSG101) in β-TM or HD exosomes (**d**) Flow cytometric analysis for characterization of the cellular origin of exosomes (RBCs-CD235a; platelets-CD40L; monocytes-CD11b; Hematopoietic progenitor cells-CD34+ and exosomal marker- CD63).

Exosomes isolated from human plasma of healthy donors (HD) and β-thalassemia major (β-TM) patients were analyzed for their size distribution using DLS (Dynamic Light Scattering) instrument (n = 10). β-TM patients had slightly large sized exosomes (70.4 ± 6.0 to 167 ± 13 nm) compared to HD exosomes (36.5 ± 3.9 to 78.7 ± 6.6 nm) (Fig 1a). All the samples had an average particle diameter of approximately 70–80 nm, which is concordant with the previous report^25^. Further, the exosome count was analyzed by flow cytometry (Fig. 1b). Compared to the HD (195.7 exosomes/μL), β-TM patients showed a higher exosome number (14238 exosomes/μL). We further characterized the exosome identity by analyzing their surface markers such as CD63 and TSG101 through western blot. Figure 1c shows exosomal marker CD63 expression in HD and β-TM exosomes, while TSG101 expression was observed in β-TM exosomes. Exosomes express specific markers with respect to their cellular origin^26^. In order to measure the cellular origin, cell surface antigens- CD235a, CD40L, CD11b, CD34 and CD63 in the exosomes were detected by flow cytometry. From the results, percentage of cell-specific exosomes in HD was found to be RBCs (31.2%; CD235a), platelets (46.25%; CD40L), hematopoietic progenitor cells (22%; CD34+) and monocytes (17.72%; CD11b). Cell-specific exosomes in β-TM was found to be RBCs (17.4%; CD235a), platelets (24.6%; CD40L), hematopoietic progenitor cells (43.3%; CD34+) and monocytes (14.5%; CD11b) (Fig. 1d). These results show that β-thalassemia major patients had increased plasma exosomes compared to healthy donor. Further, HD and β-TM exosomes had majority of their cellular origin from platelets and CD34+ hematopoietic progenitor cells respectively.

### Exosomal miRNA profiling by next-generation sequencing (NGS) analysis

**Figure 2:**
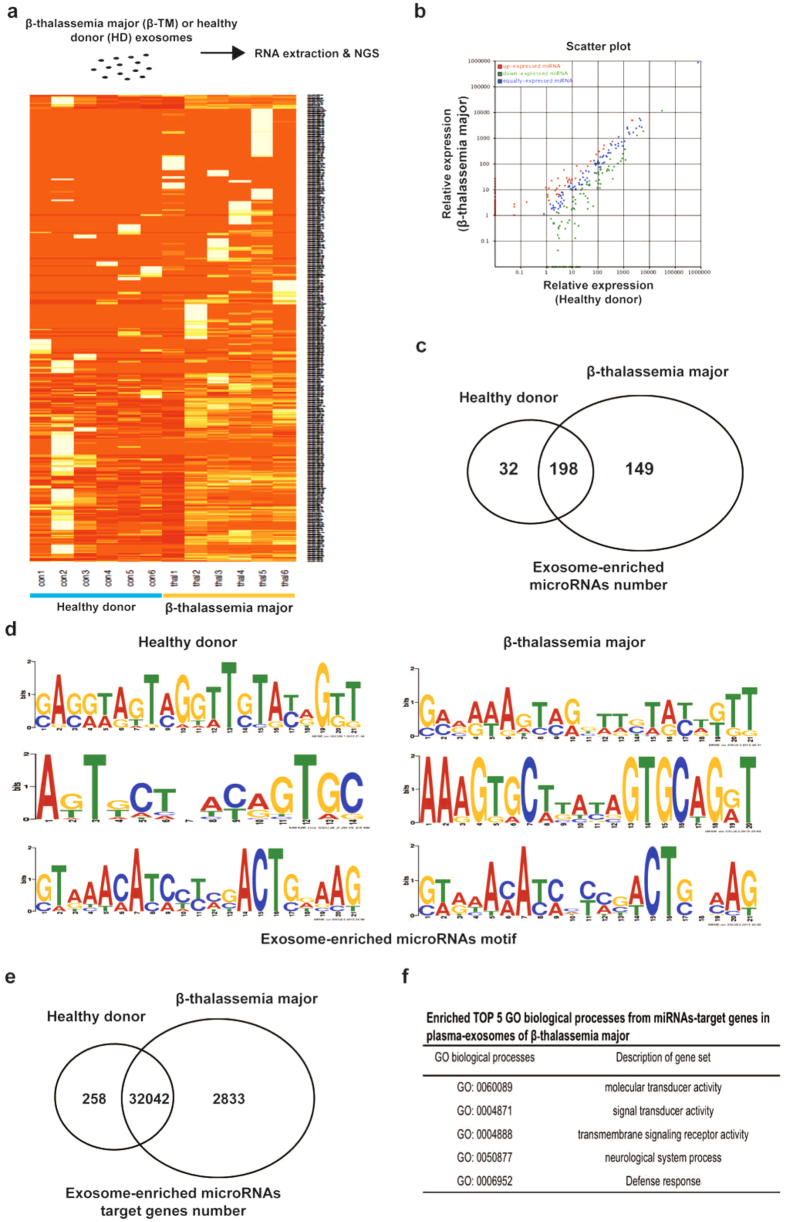
miRNA and bioinformatic analysis of healthy donor and β-thalassemia major exosomes. (**a**) Heatmap showing hierarchical clustering of differentially expressed miRNA levels from plasma exosomes of β-TM and HD by next-generation sequencing. (**b**) Scatter plot matrix of differentially expressed miRNAs in HD (x-axis) and β-TM (y-axis) by NGS analysis. (**c**) Counts in the Venn diagram are the number of exosome-enriched miRNAs identified by NGS in HD and β-TM. (**d**) Top three sequence motifs significantly enriched in exo-miRNAs (MEME tool). (**e**) Counts in the Venn diagram are the number of target genes for exosome-enriched miRNAs identified by NGS in HD and β-TM. (**f**) Gene ontology analysis of target genes from exosome-enriched miRNAs showing top 5 biological processes in β-TM.

To characterize the global miRNAs changes that occur in plasma-exosomes, we performed NGS in 12 samples (Twelve RNA samples were obtained from β-TM patients (n = 40) and HD (n = 40) by pooling RNA from six or seven patients within one group as ‘one sample’). Differential miRNAs expressions provide information on the miRNAs involved in disease regulation. Data analysis of hierarchical clustering showed differential miRNA expression in the two groups (Fig. 2a,b). 32 and 149 miRNAs were uniquely expressed in HD and in β-TM patients respectively, whereas 198 miRNAs were found to express in both groups (Fig. 2c). An impartial search for specific motifs in exo-miRNA was identified using MEME analysis (http://meme-suite.org). Figure 2d shows three differentially represented motifs in HD or β-TM patients. We further identified the possible genes altered by the exo-miRNAs using Gene Ontology Consortium (http://geneontology.org). Bioinformatics prediction showed that exo-miRNA from the β-TM patients and HD could alter 2833 and 258 genes respectively. While 198 exo-miRNAs expressed in both β-TM and HD had targets for 32042 genes. Differentially regulated miRNAs from β-TM patients exosomes have been predicted to regulate broad range functions including; molecular and signal transducer activity, transmembrane signaling receptor activity, neurological system process and defense response (Fig. 2e,f). Thus, β-TM patients have plasma exosomes with a lot of differentially expressed miRNA profile, which might potentially involve in various biological processes.

### miR-223-3p targets 3′UTR of LMO2 and down regulates γ-globin expression

**Figure 3:**
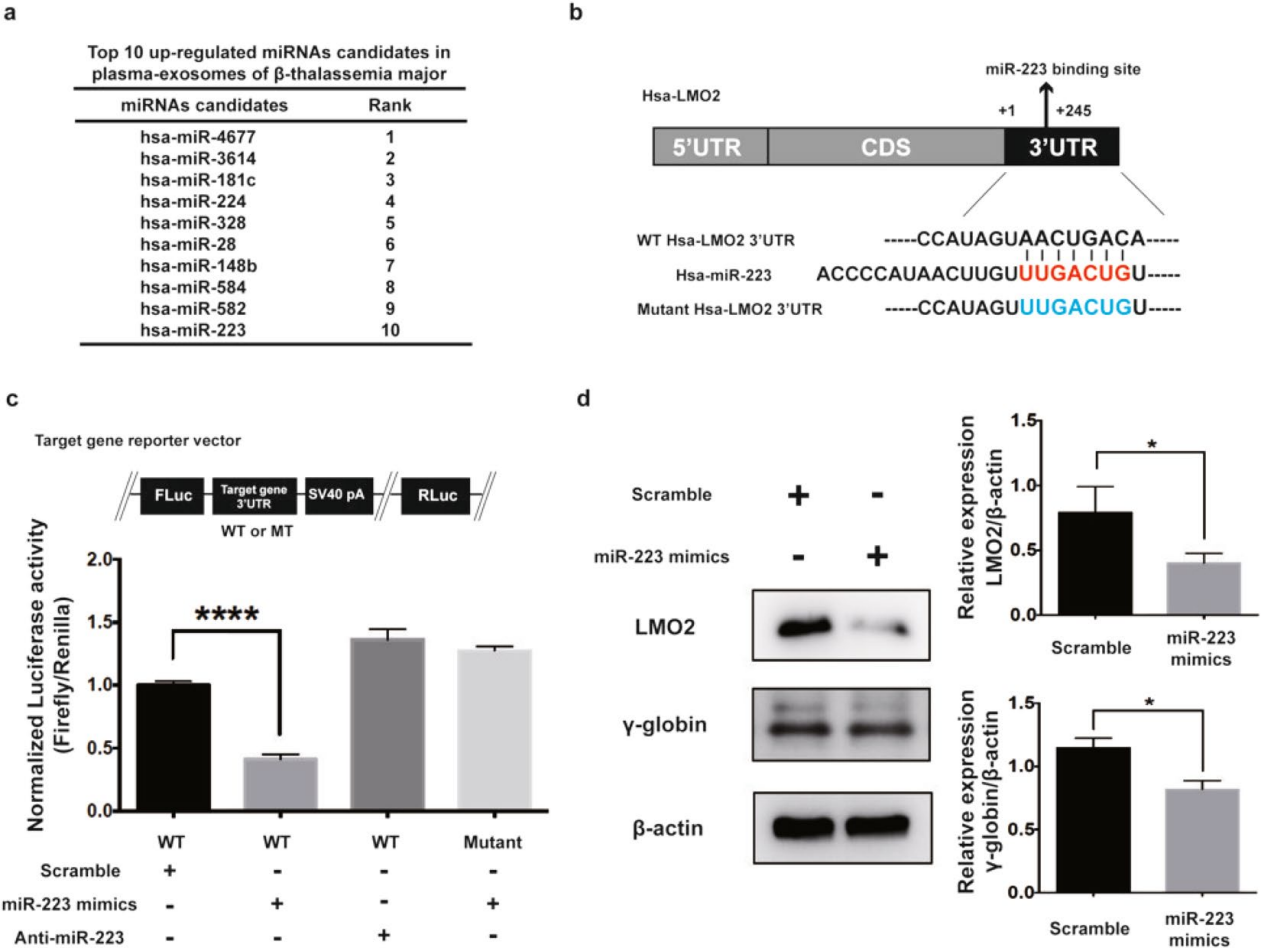
miR-223-3p down regulates LMO2 and γ-globin expression. (**a**) Top10 up-regulated miRNA candidates in the plasma exosomes of β-TM as determined by NGS analysis. (**b**) Predicted miR-223-3p binding site in the 3′UTR of LMO2 with sequence complementarity. (**c**) Schema of a reporter vector (pmirGLO) carrying either WT or mutant 3′UTR of LMO2. Luciferase assays using reporter vector (pmirGLO) carrying either WT or mutant 3′UTR of LMO2 in K562 cells transfected with scramble control microRNA mimics (controls, 100 nM), miR-223-3p mimics (100 nM) or anti-miR-223-3p (antagomir, 100 nM) for 24 hr. Experiments were performed in triplicates. The values represented are mean ± SD, one-way ANOVA, ****P < 0.0001, Prism6 (GraphPad Software Inc.). (**d**) Immunoblot of LMO2 and γ-globin expression in the presence of miR-223-3p mimics in K562 cells. Experiments were performed in triplicates. The values represented are mean ± SD, t-test analysis, *P < 0.05, Prism6 (GraphPad Software Inc.).

From the NGS data, we shortlisted top 10 significantly up regulated miRNAs (miR-4677, miR-3614, miR-181c, miR-224, miR-328, miR-28, miR-148b, miR-584, miR-582 and miR-223) (>2.0 fold with p < 0.05) among 198 plasma exo-miRNAs enriched in HD and β-TM (Fig 3a). Among them, miR-223-3p was reciprocally expressed in HD and β-TM exosomes. We then analyzed the target gene for miR-223-3p with functional relation to γ-globin activation or repression in the 32042 genes regulated by exo-miRNAs using TargetScan (http://www.targetscan.org), miRanda database, (http://www.microrna.org/microrna/getMirnaForm.do) and RNAhybrid (https://bibiserv2.cebitec.uni-bielefeld.de/rnahybrid). From the previous studies^27^ and from our bioinformatics analysis, we predicted miR-223-3p might bind to 3′UTR of LMO2 (ENST00000395833.3), an important regulator of erythroid differentiation (Fig. 3b). We then cloned the 3′UTRs of LMO2 and their seed-sequence-mutated versions into downstream of the open reading frame (ORF) of a firefly luciferase reporter gene and assessed the ability of miR-223 to down regulate luciferase expression. MiR-223-3p bound to wild-type 3′UTR of LMO2 and decreased firefly luciferase activity. MiR-223-3p did not bind to the mutated LMO2 3′UTR (Fig. 3c). Further, treatment with miR-223-3p mimics significantly down regulated LMO2 and γ-globin expressions in K562 cells (Fig. 3d). Thus, miR-223-3p down regulates γ-globin expression through LMO2 targeting.

### miR-138-5p targets 3′UTR of BCL11A and up regulates γ-globin expression

**Figure 4:**
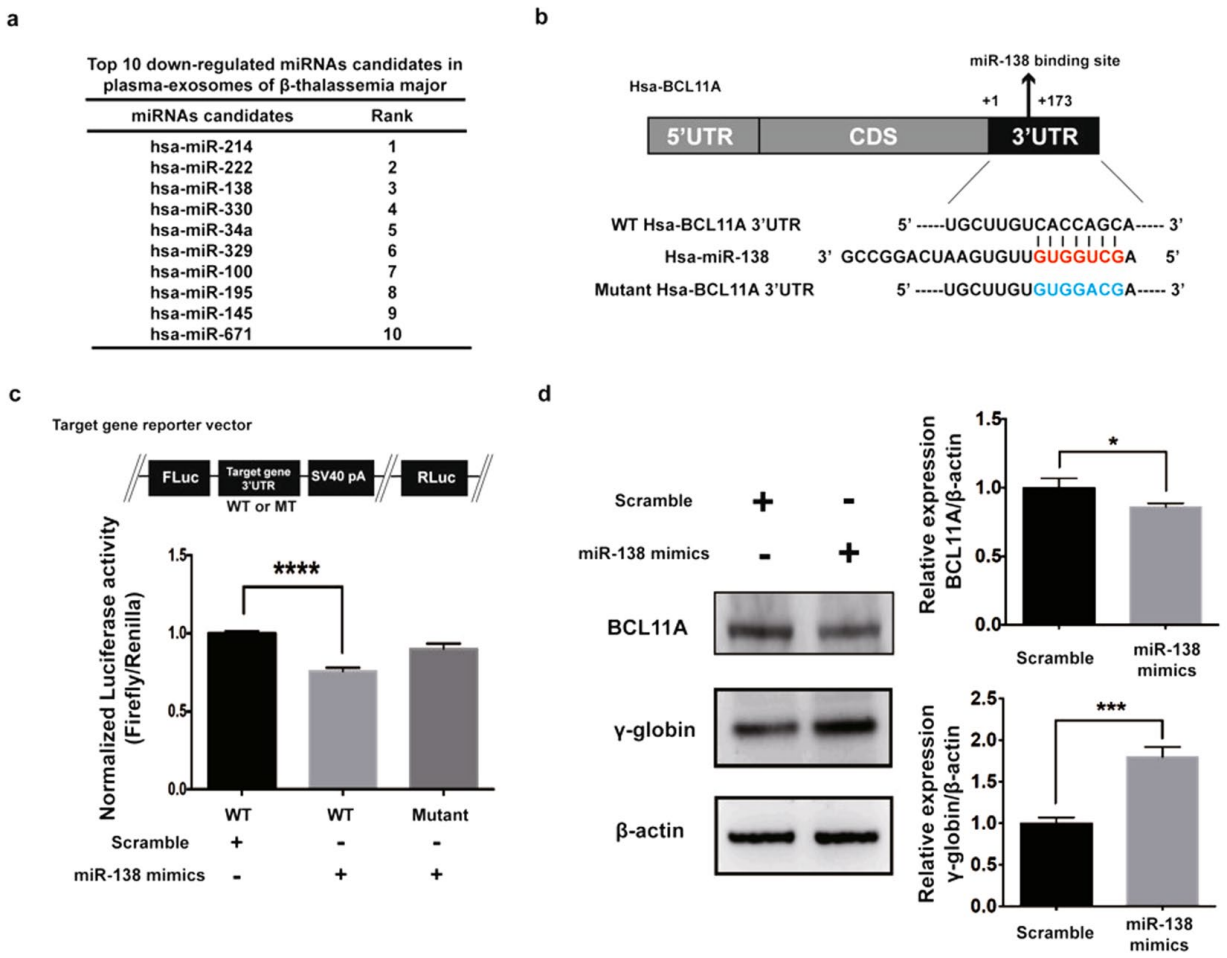
miR-138-5p targets BCL11A and regulates γ-globin expression. (**a**) Top10 down-regulated miRNA candidates in the plasma exosomes of β-TM as determined by NGS analysis. (**b**) Predicted miR-138-5p binding site in the 3′UTR of BCL11A with sequence complementarity. (**c**) Schema of reporter vector (pmirGLO) carrying either WT or mutant 3′UTR of BCL11A. Luciferase assays using a reporter vector (pmirGLO) carrying either WT or mutant 3′UTR of BCL11A in K562 cells transfected with scramble control microRNA mimics (controls, 100 nM), miR-138-5p mimics (100 nM) for 24 hrs. Experiments were performed in triplicates. The values represented are mean ± SD, one-way ANOVA, ****P < 0.0001, Prism6 (GraphPad Software Inc.). (**d**) Western blot of BCL11A and γ-globin expression in the presence of miR-138-5p mimics in K562 cells. Experiments were performed in triplicates. Values represented are mean ± SD, t-test analysis, *P < 0.05, Prism6 (GraphPad Software Inc.).

Figure 4a shows top 10 significantly down regulated miRNAs (<0.5 fold with p < 0.05) (miR-214, miR-222, miR-138, miR-330, miR-34a, miR-329, miR-100, miR-195, miR-145 and miR-671) among 198 exo-miRNAs shortlisted from NGS analysis. We found that miR-138-5p was reciprocally expressed in HD and β-TM exosomes. Further, target gene for miR-138-5p with the functional relation to γ-globin activation or repression in the 32042 genes regulated by exo-miRNAs was determined through TargetScan, miRanda database, and RNAhybrid. We found that miR-138-5p showed a specific target for BCL11A (ENST00000356842), an HbF silencer (Fig. 4b). Reporter assay was carried out to determine whether miR-138-5p regulates BCL11A expression. For this, BCL11A 3′UTRs and their seed-sequence-mutated versions were cloned into downstream of the open reading frame (ORF) of a firefly luciferase reporter gene and assessed the ability of miR-138-5p mimics on BCL11A expression. MiR-138-5p bound to wild-type 3′UTR of BCL11A but not to the mutated form and decreased the firefly luciferase activity (Fig. 4c). Figure 4d shows miR-138-5p mimics on BCL11A and γ-globin expressions in K562 cells. miR-138-5p significantly down regulated BCL11A and up regulated γ-globin expressions compared to scramble control. Thus, miR-138-5p regulates BCL11A and γ-globin expressions.

### HD and β-TM exosomes regulate γ-globin expression through BCL11A and LMO2 targeting

**Figure 5:**
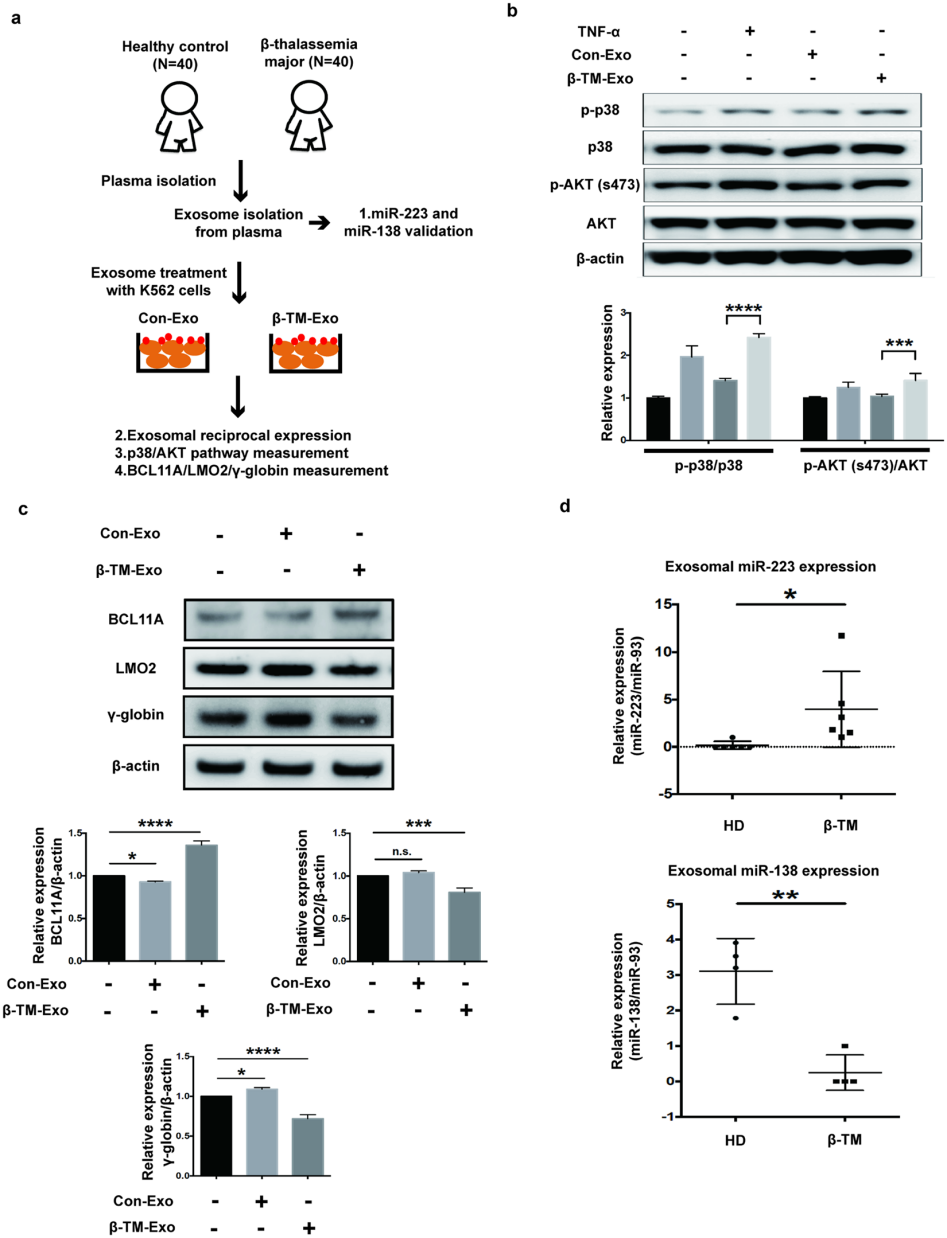
Exosomes reciprocally regulate γ-globin expressions. (**a**) Schematic representation of exosome isolation, exo-miRNA validation and γ-globin regulation. (**b**) Immunoblot analysis of p-AKT, p-p38 levels in K562 cells treated with β-TM or HD plasma exosomes. Experiments were performed in triplicates. The values represented are mean ± SD, one-way ANOVA, ***P < 0.001, ****P < 0.0001, Prism6 (GraphPad Software Inc.). (**c**) Western blot of LMO2, BCL11A and γ-globin expressions in K562 cells treated with HD and β-TM exosomes. The values represented are mean ± SD, one-way ANOVA, *P < 0.05, ***P < 0.001, ****P < 0.0001, Prism6 (GraphPad Software Inc.). (**d**) RT-qPCR analysis for plasma exo-miR-223-3p and plasma miR-138-5p expressions in HD (n = 15) and β-TM (n = 20). Data are presented as scatter plots, and the line represents the mean. Experiments were performed in triplicates. The values represented are mean ± SD, two-way ANOVA, *P < 0.05, **P < 0.01, Prism6 (GraphPad Software Inc.).

Schematic representation of HD and β-TM exosome isolation and analysis of exosome-mediated signaling in K562 cells (Fig. 5a). Signaling pathway of Akt and MAPK are key regulators of cellular proliferation^28^. In order to determine exosome-induced cell signaling, K562 cells were treated with exosomes (10 ul) isolated from either HD or β-TM plasma (200 ul) and then analyzed for Akt and p38 phosphorylation. TNF-α treatment was used as positive control. From the results, β-TM exosomes significantly up regulated p-Akt and p-p38 levels compared to HD (Fig. 5b). We next analyzed the functional role of HD and β-TM exosomes on LMO2, BCL11A and γ-globin expressions in K562 cells. Exosomes from healthy donor down regulated BCL11A expression and up regulated γ-globin expression. Whereas, β-TM exosomes down regulated both LMO2 and γ-globin expressions (Fig. 5c). Further, we validated exo-miR-223-3p and exo-miR-138-5p expression level in HD (n = 15) and β-TM patients (n = 20) using RT-qPCR. Samples with non-determined levels of miRNA expressions were excluded. The results showed that exo-miR-223-3p and exo-miR-138-5p were reciprocally expressed in β-TM and HD. Significantly higher exo-miR-223-3p expression was observed in β-TM compared to HD while exo-miR-138-5p expression was higher in HD compared to β-TM (Fig. 5d). Thus, exosomes from HD and β-TM patients reciprocally regulate γ-globin expression possibly through their differential exo-miRNA expressions.

### Repression of miR-223-3p up-regulates γ-globin expression

**Figure 6:**
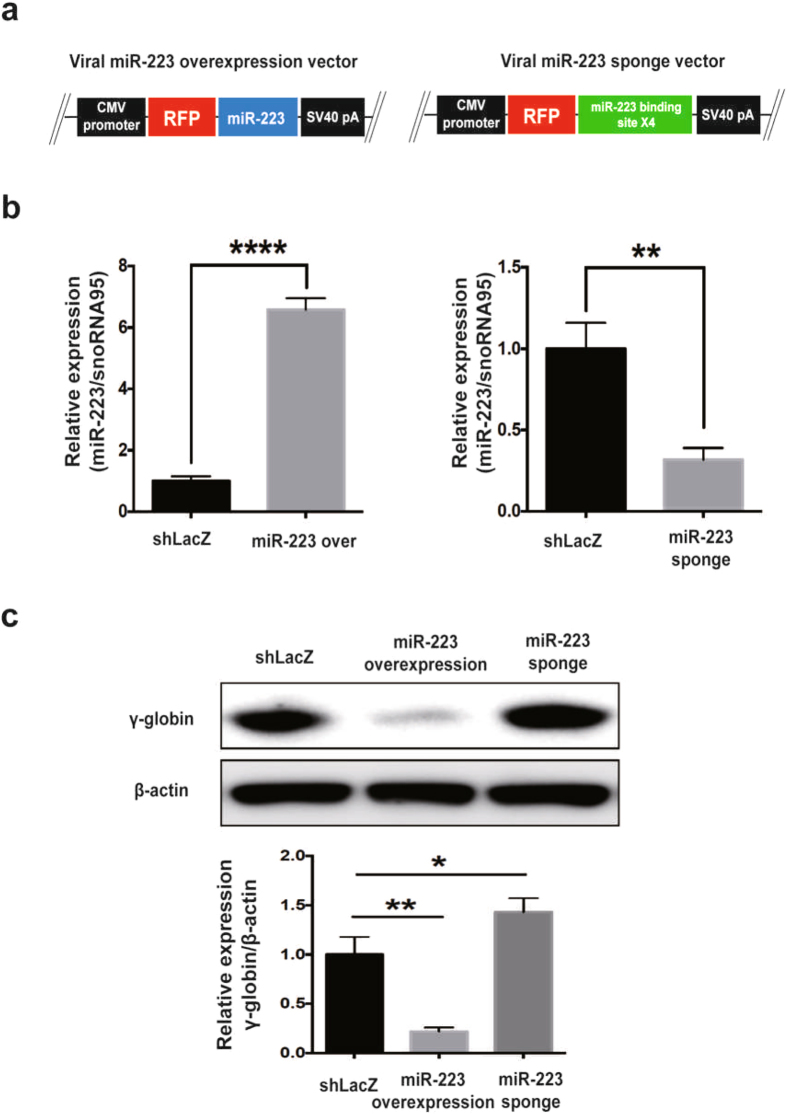
miR-223-3p targeting up regulates γ-globin expression. (**a**) Overview of the barcoded lentiviral miRNA over expression and sponge vector. miRNA expression is driven by the viral CMV promoter. This vector contains the miRNA and ~500 bp endogenous flanking sequences in the 3′UTR of the RFP (Red Fluorescent Protein). To allow selection of target cells, we incorporated a dual selection cassette of puromycin gene. (**b**) RT-qPCR validation for miR-223-3p over expression and sponge repression in K562 cells. (**c**) Western blot analysis of γ-globin expression by viral miR-223-3p over expression or sponge repression in K562 cells. Experiments were performed in triplicates. The values represented are mean ± SD, t-test analysis, *P < 0.05, **P < 0.01, ****P < 0.0001, t-test, Prism6 (GraphPad Software Inc.).

Lentiviral-mediated miRNA over expression and sponge repression is an approach to study gain or loss of function^29, 30^. We constructed miR-223-3p viral over-expression and sponge vector, in order to confirm miR-223-3p mediated γ-globin expression. Figure 6a shows miR-223-3p over expression and miR-223-3p sponge vector construct. MiR-223-3p expressions were determined through RT-qPCR to confirm miR-223-3p over expression and sponge repression in K562 cells (Fig. 6b). Cells transfected with miR-223-3p over expression vector showed dramatic suppression of γ-globin expression; however, cells transfected with miR-223-3p sponge vector resulted in re-activation of γ-globin expression (Fig. 6c). These results reveal that specific miR-223-3p targeting might re-activate γ-globin expression.

### hnRNPA1 affects exosomal miR-223-3p levels

**Figure 7:**
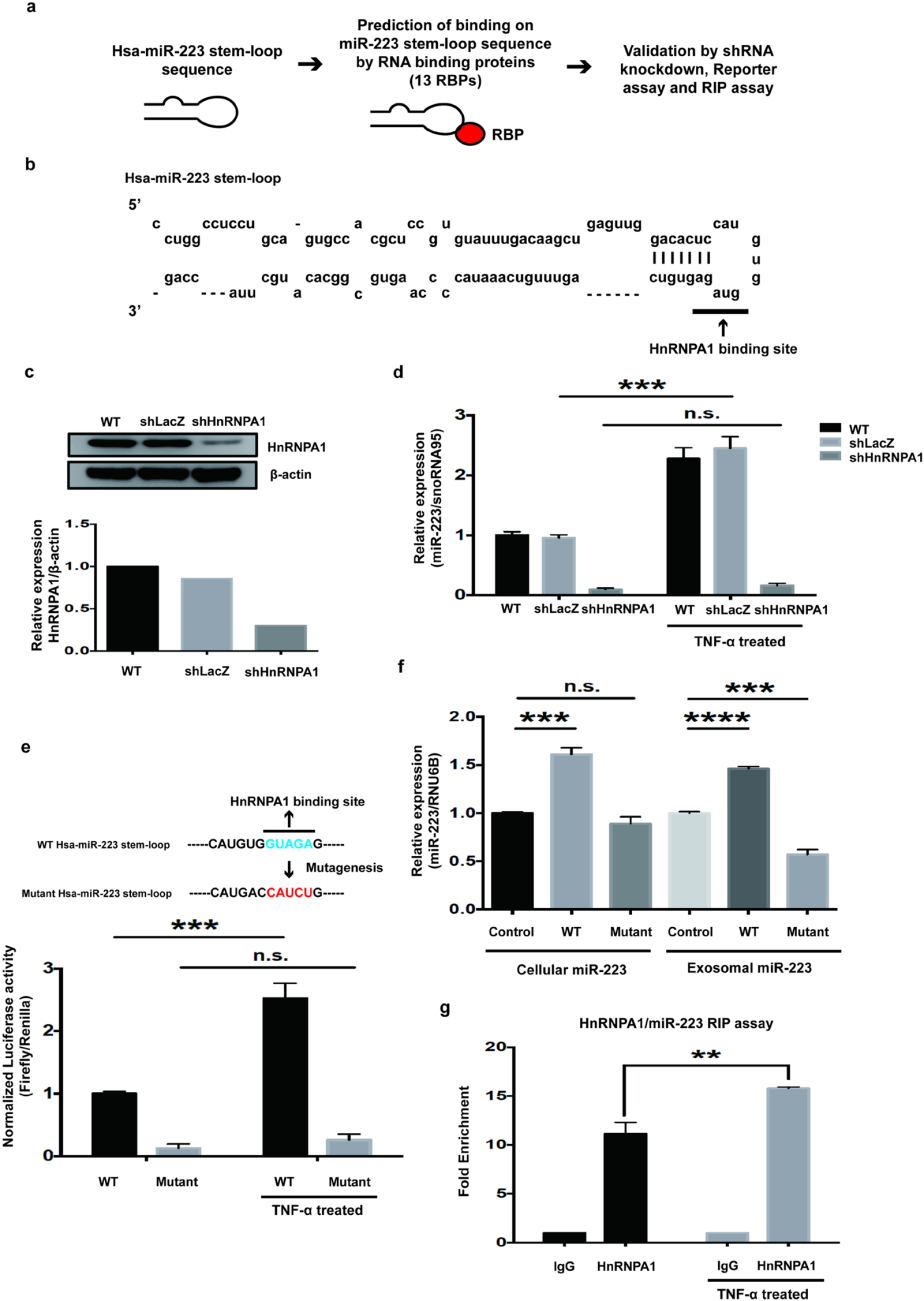
hnRNPA1 affects exo-miR-223-3p levels. (**a**) Schematic representation for identification of RBP mediated miR-223 recognition (**b**) hnRNPA1 motif (GUAGUAGU) binds to stem-loop structure of pre-miR-223 as determined by RBP map computational tool. (**c**) K562 cells were transfected with shRNAs against hnRNPA1 or control LacZ. Densitometric analysis of western blots shows successful shhnRNPA1 knockdown (KD). (**d**) miRNA RT-qPCR analysis of exo-miR-223-3p levels in K562- shhnRNPA1-KD or shLacZ cells in the presence/absence of TNF-α treatment for 24 hrs. (**e**) Schematic representation of viral pLAS2 vector and wild-type (WT) or mutant (MT) hnRNPA1 binding site in stem-loop miR-223 sequence. Reporter assay in K562 cells transfected with miR-223-WT or -MT and with/without TNF-α treatment for 24 hrs. (**f**) Cellular and exo-miRNA-223-3p levels in TNF-α induced K562 cells in the presence of wild-type (WT) or mutant (MT) stem loop miR-223-3p vector. (**g**) RNA immunoprecipitation assay for miR-223-3p was performed with anti-hnRNPA1 or IgG as a control antibody in K562 cells treated with or without TNF-α. Experiments were performed in triplicates. The values represented are mean ± SD, two-way ANOVA, **P < 0.01, ***P < 0.001, ****P < 0.0001, Prism6 (GraphPad Software Inc.).

Previous results show that, increased exo-miR-223-3p levels in β-TM is involved in γ-globin silencing. So, we combined bioinformatics, shRNA-mediated RBP (RNA binding protein) knockdown, miR-223-3p stem-loop luciferase-based reporter assay and RNA immunoprecipitation to understand how miR-223-3p is increased in the exosomes. *In vitro* studies were carried out in K562 cells (human erythroleukemia cell line) in the presence/absence of TNF-α. TNF-α mediated inhibition of erythropoiesis have been previously reported^31–33^. Next, we identified the specific interaction between pre-miR-223 stem and loop sequence and motifs of 13 hnRNP family proteins (Supplementary Table S1) through RBP map analysis. Results showed that hnRNPA1 motif had specific binding to pre-miR-223 (Fig. 7a,b). In order to test the implication and identify the exact function *in vitro*, first we successfully performed heterogeneous nuclear ribonucleoprotein A1 (hnRNPA1) knockdown in K562 cells (Fig. 7c). Next to identify whether hnRNPA1 has a regulatory role in exo-miR-223 levels; WT, shLacZ, and shhnRNPA1 cells were treated with TNF-α or vehicle and analyzed for miR-223-3p expression using RT-qPCR. The results show that WT and shLacZ cells showed significantly increased levels of miR-223-3p levels after TNF-α treatment. However, shhnRNPA1 knockdown cells show low expression of miR-223-3p levels. In addition, shhnRNPA1 cells treated with TNF-α did not increase exo-miR-223-3p levels compared to shLacZ cells (Fig. 7d). Finally, to test the implications of miRNA recognition by hnRNPA1, we used a luciferase-based reporter assay. The WT or mutant precursor stem-loop of miR-223-3p was cloned downstream of the firefly luciferase ORF, and the plasmid was transiently transfected into K562 cells. Upon TNF-α treatment, cells transfected with the mutant recognition sequence of the stem-loop of miR-223-3p plasmid showed decreased luciferase expression compared to the WT-miR-223-3p transfected plasmid (Fig. 7e). In order to evaluate whether miR-223 mutation affects exosomal miR-223-3p levels, we transfected WT and mutant stem-loop miR-223-3p in TNF-α induced K562 and checked for cellular and exo-miR-223-3p levels. WT miR-223-3p showed significantly increased levels of cellular miR-223-3p expression compared to control, while mutant-miR-223-3p had non-significant miR-223-3p levels. Further, exo-miR-223-3p levels were significantly higher in WT miR-223-3p while miR-223-3p mutagenesis showed significantly decreased exo-miR-223-3p expression compared to control (Fig. 7f). Then, we evaluated the direct interaction between hnRNPA1 and miR-223-3p by RNA immunoprecipitation (Fig 7g). We found that miR-223-3p was enriched with the hnRNPA1 antibody compared to IgG (control antibody). In addition, TNF-α induced K562 cells showed significant miR-223-3p enrichment compared to control cells. Taken together, the results show that hnRNPA1 is involved in miR-223-3p recognition and affects exo-miR-223-3p levels.

## Discussion

A developmental hemoglobin switch occurs between α and β-globin-like clusters (ε, β, δ, γ) from embryonic to adult stage, where HbA is the predominant form in adults. Mutation in β-globin gene complex produces functionally defective β-globin chains leading to a severe anemic condition in β-thalassemia, one of the major clinical subtypes of hemoglobinopathies^2, 3^. An effective therapeutic approach to treat β-thalassemia could be achieved by re-activating developmentally silenced γ-globin to produce functional HbF. Here, we show for the first time that, exo-miRNAs might regulate γ-globin expression. Exosomes and exo-miRNAs (miR-223-3p and miR-138-5p) reciprocally regulate γ-globin expression and their differential levels are highly associated with β-TM. Further, miR-223 targeting up regulates γ-globin expression.

Exosomes are nanovesicles released into the body fluids by diverse cell types and mediate cell-cell communication. Exosomes regulate normal cellular processes; however, under pathological conditions increased levels of exosomes are secreted^34^. In β-thalassemia patients, significantly higher levels of RBC vesicles^24^ and platelet-derived micro particles^23^ were attributed to the thromboembolic complications. Higher microparticle release in β-thalassemia is associated with cardiovascular complications including pulmonary hypertension, arterial stiffness, and vascular dysfunction^35^. Erythroid cells derived from CD34+ HPCs reported to have miRNA-mediated control on the HbF expressions in β-thalassemia^14^. Further, apoptosis of erythroid progenitors CD34+ in β-thalassemia is linked to immature RBCs production and ineffective erythropoiesis. In this report, we identified that β-TM patients had higher levels of plasma exosomes and the majority of them were derived from CD34+ hematopoietic progenitor cells compared to that of HD. Thus, increased exosome release from CD34+ progenitor cells might have an important regulatory role in β-TM.

Cell and stimuli specific miRNAs are synthesized and packaged into exosomes to mediate miRNA-based intercellular communication^36^. Highly enriched exo-miRNAs might act as disease biomarkers and significantly impact on the target cells^21^. In this study, we demonstrate global differential miRNA expression in plasma exosomes of β-TM compared to HD. Nearly, 149 miRNAs were uniquely expressed in β-TM exosomes while HD exosomes contained 32 miRNAs. Selective exo-miRNA expressions under a pathological condition depends on their sorting into the exosomes, which is regulated through miRNA motif recognition^37^. We identified that exo-miRNAs from HD and β-TM displayed differential motifs, which might be important for their exosomal loading. Thus, exo-miRNAs under normal and β-TM might mediate target gene regulation through intercellular communication.

Deregulated miRNA expressions act as disease biomarkers and miRNAs regulate gene expression through post-transcriptional silencing^16, 17^. γ-globin activators and repressors are identified as potential targets for inducing HbF expressions^38, 39^. Previously, studies have demonstrated on miRNA-mediated regulation of HbF expressions. MiR-96 directly represses γ-globin expression and negatively regulates HbF levels^40^. Persistence of HbF and delayed fetal-to-adult haemoglobin switching in human trisomy 13 showed miR-15a/16-1 mediated down regulation of MYB^9^. Here, we identified firstly that, plasma exosomes and exo-miRNAs controls γ-globin expressions in K562 cells and their differential levels have an impact on β-TM. A severe anemic condition in thalassemia activates erythropoietin (EPO) induced erythroid hyperplasia. EPO-induced erythropoiesis is regulated via (JAK2/STAT5), (PI3K)/AKT and (MAPK/ERKs) signaling pathways^41–43^. However, the erythroid precursor expansion through the feedback loop mechanism remains unsuccessful due to ineffective erythropoiesis. Previous studies demonstrate that increased erythroid precursor cells in β-thalassemia patients^44, 45^. We found that β-TM exosomes upregulated p-AKT and p-p38 levels compared to HD exosomes. These observations might relate to exosome-mediated erythroid expansion; however, detailed studies are required to confirm the exact mechanism. Thus, among the deregulated exo-miRNAs in β-TM and HD; miR-223-3p and miR-138-3p had targets for γ-globin upstream regulators LMO2 and BCL11A respectively. Thus, differential exo-miRNA expressions might have an important regulatory control on HbF levels in HD and β-TM.

Some of the known γ-globin repressors include BCL11A, KLF1, TR2/TR4, Oct-1, MYB (reviewed)^46^. Mi2β, belonging to NuRD complex regulates γ to β-globin switching by up regulating *KLF1* and *BCL11A* genes^47^. BCL11A regulates haemoglobin switching by acting as γ-globin transcriptional repressor and reports show elevated HbF levels under BCL11A knockdown^9, 10^. KLF1 controls γ to β switching through regulating BCL11A expression^15^. BCL11A along with co-ordinated action of SOX6 binds to LCR and silences γ-globin expression^48^. Lulli *et al.* ^14^, demonstrated that hereditary persistence of HbF in β-thalassemia patients might be linked to miR-486-3p suppressive effects on BCL11A. In this study, we show that significantly up regulated miR-138-5p in HD exosomes had a specific target for BCL11A. We confirmed that miR-138 mimic and HD exosomes suppressed BCL11A expression and up-regulated γ-globin expression in K562 cells. These results suggest that γ-globin up regulation in HD might be regulated through miR-138 mediated BCL11A suppression. Thus, miR-138-5p down regulation in β-TM might relieve BCL11A suppression leading to γ-globin silencing.

Globin gene regulation is mediated through multimeric protein complex including GATA1, TAL1, KLF1 and adapter proteins (LDB1 and LMO2). The protein complex regulates globin gene expression by forming chromatin looping between LCR and globin gene promoters^4–6, 49^. Among the deregulated miRNAs in the β-TM exosomes, significantly up regulated miR-223-3p had specific target for LMO2. LMO2 is identified as an important regulator of erythropoiesis^50^. Significantly low levels of miR-223 during erythroid differentiation and maturation, regulates normal erythropoiesis by preventing LMO2 suppression^27^. Differential miR-223 levels involve in the regulation of megakaryocyte and erythroid differentiation^51^. We demonstrate for the first time that, β-TM exosomes have significantly higher levels of miR-223 compared to HD exosomes. Both β-TM exosomes and miR-223 mimic specifically targeted LMO2 and down regulated γ-globin expressions in K562 cells. Further, repression of miR-223 led to activation of γ-globin expression. These results reveal that exosome and exo-miR-223-3p mediated target gene regulation and γ-globin silencing might be clinically relevant to β-TM major. We here demonstrate that intercellular communication of exosomes and their differential exo-miRNA levels in HD and β-TM major reciprocally regulate γ-globin expressions.

Exo-miRNA mediated cell-cell communication and their target gene regulation is tightly controlled through miRNA loading into exosomes. Heterogeneous nuclear ribonucleoproteins (hnRNPs), contain RNA recognition motifs which mediate transcriptional control, RNA metabolism, importantly in miRNA biogenesis and its exosomal packaging^37, 52^. hnRNPA2B1 mediated miRNA sorting and exosomal packaging is regulated through sequence-specific recognition motifs and their directed mutagenesis enables the modulation of miRNA cargo into the exosomes^37^. Here, we demonstrate that hnRNPA1 might be a positive regulator in increasing the exo-miR-223-3p levels. hnRNPA1 is a nucleo-cytoplasmic shuttling protein binds to sequence-specific RNA and involve in miRNA processing^53^. hnRNPA1 specifically interacts with primary RNA and mediates regulation of miR-18a biogenesis and contributes to its functional role^54^. Michlewski and Caceres, have reported on the antagonistic role of hnRNPA1 in let-7A biogenesis. The study demonstrates that DROSHA-mediated pre-let-7A-1 processing was inhibited by hnRNPA1 binding to its conserved terminal loop^55^. In the present study, we found that hnRNPA1 specifically bound to the stem-loop structure of miR-223-3p. Further, hnRNPA1 knockdown or miR-223 mutagenesis in the stem-loop structure resulted in less mature exo-miR-223-3p levels. Thus, regulation of exosomal miR-223-3p levels by hnRNPA1 might be critical to its target gene regulation in β-TM.

**Figure 8:**
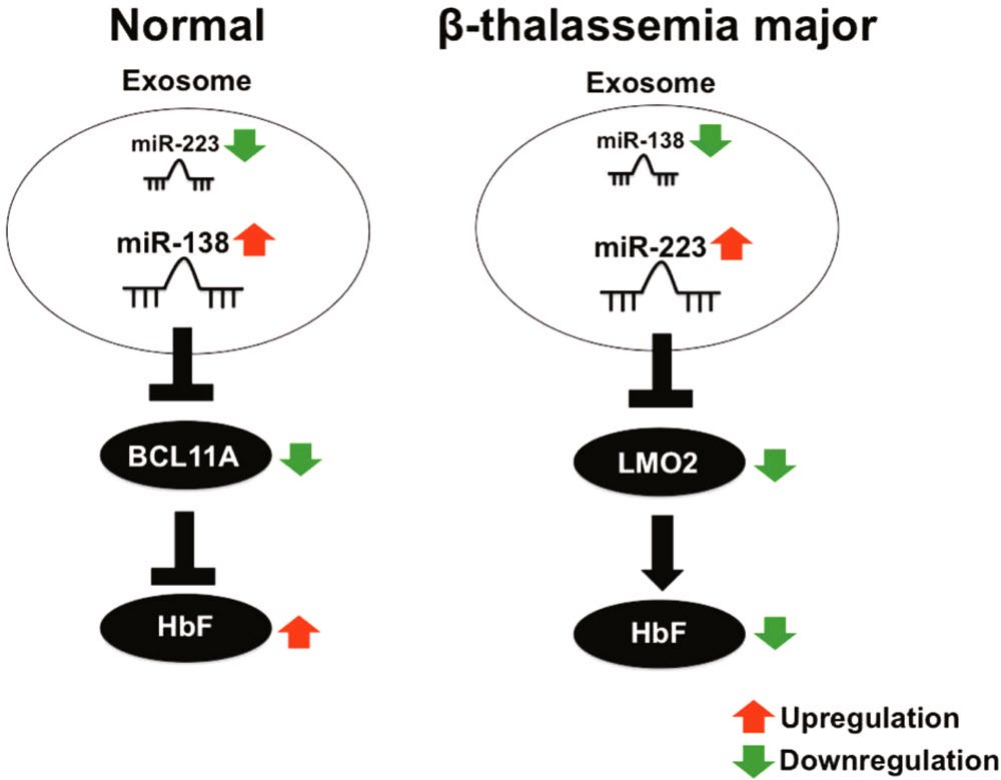
Exo-miRNA mediated γ-globin regulation. Reciprocally expressed exo-miRNAs (miR-223 and miR-138) in the healthy donor and β-TM are involved in the γ-globin regulation.

In summary, our findings demonstrate for the first time that plasma exo-miRNAs; miR-223-3p and miR-138-5p reciprocally regulates γ-globin expressions by targeting LMO2 and BCL11A expressions respectively. The study shows evidence that the differential levels of exo-miRNAs are involved in γ-globin silencing in β-TM. Further, hnRNPA1 is involved in miR-223-3p recognition and affects its exosomal levels. Figure 8 shows the schematic representation of exo-miRNA mediated γ-globin regulation. Collectively, the study unravels exo-miRNA mediated γ-globin regulation and hnRNPA1-exo-miR-223-LMO2 axis may be critical to γ-globin regulation in β-thalassemia major.

## Materials and Methods

### Patients and blood samples

Blood samples were collected from β-thalassemia major patients (n = 40) ranging in age from 12 to 41 years. Control samples were collected from healthy and age-matched individuals (n = 40). All subjects had no evidence of concurrent infection for past 6 months and none had been hospitalized. After obtaining written informed consent, 25 ml of venous blood was collected by venipuncture and aliquoted equally into BD Vacutainer® tubes containing ACD solution. All blood samples were collected at room temperature (24-26°C) and processed within 4 h. Blood samples were centrifuged at 1500 g for 10 mins and the supernatant containing plasma was stored in aliquots at −80 °C until further analysis. Standard hematological techniques and Hb analysis were used for diagnosis of β-thalassemia major. All the subjects were analyzed and confirmed for normal glucose-6-phosphate dehydrogenase levels. The study was approved by the Research Ethics Committee of China Medical University & Hospital (CMUH104-REC1-059). The methods were carried out in accordance with the approved guidelines.

### Exosome isolation

Plasma exosomes were isolated using exosome isolation kit (Invitrogen). Briefly, plasma samples were centrifuged at 2000 × *g* for 20 mins to remove cell debris. The supernatant was again centrifuged at 10,000 × *g* for 20 mins and the resulted pellet was discarded. The plasma was diluted with PBS and the mixture was vortexed thoroughly. To this, exosome precipitation reagent was added and incubated for 10 mins. The samples were centrifuged at 10,000 × *g* for 5 minutes and the pellet containing exosomes were re-suspended in PBS, RIPA buffer, or Qiazol depending on the experimental procedure. Limitation of the present study is that the isolated exosomes might prone to contain HDL, LDL, and other small sized particles.

### Exosome size analysis

Size analysis of exosomes was performed by dynamic light scattering (DLS) technique using a Zetasizer Nano ZS (Malvern Instruments, UK) according to the manufacturer’s instructions. Briefly, exosomes (10 µl) were diluted in PBS to a final volume of 100 µl and three-80 sequential acquisitions were performed for each exosomal preparation.

### Exosome characterization using flow cytometry

The isolated exosomes were re-suspended in phosphate buffered saline (PBS; 18.6 mM NaH_2_PO_4_H_2_O, 84.1 mM Na_2_HPO_4_, 1.5 M NaCl) containing 1% heat-inactivated fetal bovine serum (FACS buffer). Flow cytometry data were collected on a FACS Canto flow cytometer using FACSDiva v8.0 software (BD Biosciences). The analysis was performed using FlowJo v10.0.5 (Tree Star, Ashland, OR.). Exosome count was analyzed after gating on the live cell population based on FSC/SSC. For cellular exosome characterization, isolated plasma-exosomes were rested at 37 °C for 15 min. Exosomes were then incubated with 10 µg/ml of primary antibody CD235a (GA-R2, CD235a-FITC, catalogue no 561017, BD Biosciences), CD40L (EP462E, CD40L, catalogue no ab52750, Abcam), CD11b (ICRF44, CD11b-APC, catalogue no 550019, BD Biosciences), CD34 (4H11, CD34-PE, catalogue no ab18228, Abcam) and CD63 surface expression (NKI/C-3, catalogue no MA5-11501, Thermo) or left untreated. The exosomes were washed twice with ice-cold FACS buffer. The exosomes were then stained with 0.25 µg of FITC-linked secondary antibody in FACS buffer for 30 mins at 4°C. Followed by washing thrice with FACS buffer, the exosomes was analyzed by flow cytometry.

### Sample collection and high-throughput sequencing of miRNAs

Plasma exosomes isolated from peripheral blood samples (20 ml) from each donor was suspended in PBS (1 ml) and 600 μl of plasma exosomes were used for exosomal RNA isolation (miRNeasy mini kit, Qiagen, USA). RNA purified was quantified using Nano Drop 2000c Spectrophotometer (Thermo Fisher Scientific. Waltham, MA) and the concentration was found to be ≧80 ng/μl with A260/280: 1.8~2.2 A260/230: ≧1.8. Twelve RNA samples were obtained from the patients (n = 40) and control donor (n = 40) by pooling RNA from six or seven patients within one group as ‘one sample’. RNA sequencing was performed using Illumina HiSeq 2000 sequencer (IlluminaInc, San Diego, CA) (BGI, Shanghai, China). The total RNAs (1 µg) were separated on 15% denaturation polyacrylamide gels and the band of small RNA fragments between 18 and 32 nt in size were excised. The size distribution and absence of rRNAs were confirmed using Agilent 2100 Bioanalyzer RNA 6000 Nano (Agilent Technologies, Palo Alto, CA.). Recovered RNAs from the gels were ligated to 59 adaptor and 39 adaptor sequentially and reverse-transcribed to cDNA to obtain sufficient product for Illumina short read sequencing technology (Hiseq 2000). Total reads obtained were between 2.2–4.8 million. The cDNA products of the small RNA fragments after sequencing will go through the data cleaning analysis and quality was checked using (FASTQC). After the removal of adapters and low-quality tags using FastqMcf and PRINSEQ, the RNA-seq data set was mapped to the human genome UCSC hg19 by SOAP or bowtie to analyze the expression and distribution in the genome. The sequences were aligned to Genbank (ftp://ftp.ncbi.nlm.nih.gov/genbank/) and Rfam- version 11 (http://rfam.janelia.org/) databases to screen and remove rRNA, scRNA, snoRNA, snRNA, tRNA associated tags and to align with non-coding sRNAs. Then the small RNA reads were annotated to miRBase database-version 21 (http://www.mirbase.org/ftp.shtml) using blast or bowtie to identify known miRNA expressions. Reads were normalized with reads per million (RPM). The reads for each miRNA were calculated for Log2 ratio and median centered values. Clustering was done by average linkage method using Pearson correlation. Differential miRNA expressions and statistical analysis were performed in R/Bioconductor using DESeq package^56^ and the miRNAs were shortlisted based on fold change (up-regulated miRNAs >2; down-regulated miRNAs <0.5) with a statistical significance of p < 0.05. Supplementary spreadsheet S1 shows the miRNA expressions from healthy donor and β-thalassemia major patients.

### Cell culture

K562 (ATCC-CCL-243) and 293T (ATCC-CRL-3216) cell lines were purchased from American Type Culture Collection. The cells were grown in Dulbecco’s modified Eagle’s medium (Gibco, Invitrogen) supplemented with 10% fetal bovine serum (FBS), 1% penicillin/streptomycin. Cells were used at 5 to 10 passages.

### Plasmids and viruses

miRNAs and reporter gene construction was performed as described previously^57, 58^. Genomic fragments of miR-223 precursors, miR-223 sponge or stem-loop constructs of miR-223 were amplified by PCR using human genomic DNA as a template. The PCR products were cloned into the pLAS2-RFP vector at restriction sites NotI and XhoI. K562 cells were infected with the virus and the expressions were then detected by using quantitative PCR (RT-qPCR). The binding site for miR-223-3p or miR-138-5p in the target genes and the entire LMO2 3′UTR or BCL11A 3′UTR sequence was cloned into the pmirGLO luciferase vector (Promega) at restriction sites PmeI and XhoI, downstream of the firefly luciferase gene. Mutation constructs were performed as described previously^59^. The mutant constructs of LMO2 3′UTR, BCL11A 3′UTR or stem-loop constructs of miR-223 were generated with a pair of primers containing the mutant sequence. All constructs were verified by sequencing. Cell transfection, reporter assays, viral production, infection and selection of transduced cells were carried out as previously described^60^.

### Exosome protein isolation

Exosomes were lysed using RIPA lysis buffer, Pierce (50 mM Tris-HCl, 150 mM NaCl, 1 mM EDTA pH 8.0, 1% Triton X-100, 1% Na-deoxycholate, 0.1% SDS supplemented with HALT protease and phosphatase inhibitor cocktail). Protein concentration was determined by Bio-Rad protein assay.

### Cell protein extraction

After treatment schedule, cells were washed with PBS and cell lysis was performed with RIPA lysis buffer (Thermo Scientific, Waltham, MA). Protein concentration was measured by Bio-Rad protein assay.

### Western blot analysis

Samples (exosomes or K562 cells) with equal protein concentration (50 μg) were subjected to SDS-PAGE and transferred to PVDF membrane (PerkinElmer, Life Science). Blots were blocked with 5% skim milk-TBS-Tween 20 for 1 h at room temperature and probed with primary antibodies against target proteins overnight at 4°C. Primary antibodies LMO2 (1:1000) (EP3257, catalogue no # GTX62229), TSG101 (1:1000) (4A10, catalogue no # GTX70255) were purchased from GeneTex. hnRNPA1 antibody (1:1000) (D21H11, catalogue no # 8443), Akt (1:2000) (C67E7, catalogue no #4691), Phospho-Akt (Ser473) (1:2000) (D9E, catalogue no #4060), p38 MAPK antibody (1:1000) (catalogue no # 9212) and Phospho-p38 (Thr180/Tyr182) antibody (1:2000) (28B10, catalogue no # 9216) were purchased from Cell Signaling. CD63 antibody (1:500) (NKI/C-3, catalogue no # MA5-11501) was purchased from ThermoScientific. γ-globin antibody was purchased from Abcam (1:1000) (EPR9708, catalogue no ab137096). β-actin antibody was purchased from Sigma-Aldrich (1:4000) (AC-15, catalogue no A5441). Followed by which blots were washed with PBST and incubated with horseradish peroxidase-conjugated secondary antibodies (1:1000) for 1 h at room temperature. The blots were washed and incubated in ECL solution (Thermo, cat. 32209) for 1 min and then exposed by ImageQuant LAS4000 (GE Healthcare).

### shRNA knockdown by viral infection

K562 cells were infected with lentivirus expressing shRNA for hnRNPA1 in the presence of 8 μg/ml protamine sulfate for 24 h, followed by puromycin (2 μg/ml; 48 h) selection. shLacZ (TRC0000231726), which targets the LacZ gene was used as a control. The knockdown efficiency of hnRNPA1 was examined using RT-qPCR and western blot.

### Prediction of miRNA target genes

The TargetScan 6.2 (http://targetscan.org), miRanda (http://www.mirorna.org/microrna/getMirnaForm.do) and RNAhybrid (https://bibiserv2.cebitec.uni-bielefeld.de/rnahybrid) databases were used to predict the miRNA binding site on the 3′UTR of LMO2 gene and 3′UTR of BCL11A gene.

### MicroRNA mimics and antagomir transfection

The miRIDIAN miRNA mimics (Dharmacon) are single-stranded chemically enhanced oligonucleotides that were designed to mimic miRNA over expression or knockdown miRNA. K562 cells were transfected with 100 nM of either the miR-223-3p or miR-138-5p mimics or scramble mimics using the Lipofectamine 2000 reagent (Invitrogen). After 24 hr, the cells were plated for the luciferase reporter assay.

### miRNA RT-qPCR for target gene validation

Plasma exosomal RNA was isolated using the miRNeasy mini kit (Qiagen, USA) following the manufacturer’s instructions or Trizol reagent for cellular RNA extraction. Real-time PCR was conducted using universal reverse primer, miRNA-specific forward primers, 2°- Master mix (Roche), and UPL probe-21 (Roche) in accordance with the manufacturer’s protocol (Applied Biosystems). Plasma exosomal miR-223-3p and miR-138-5p expressions were determined using TaqMan miRNA assays (Thermo Fisher) and the plasma invariant miRNA (miR-93-5p) was used for normalization. The miR-223-3p expressions in the *in vitro* studies were normalized to endogenous controls RNU6B/snoRNA95.

### RNA-binding protein immunoprecipitation (RIP)

RNA-binding protein immunoprecipitation (RIP) was performed using a Magna RIP RNA-Binding Protein Immunoprecipitation Kit (Millipore). Briefly, K562 cells treated with or without TNF-α were harvested using RIP lysis buffer. Then cell lysate was immunoprecipitated with the hnRNPA1 antibody (Cell Signaling, #8443) or immunoglobulin G [IgG] control with protein G magnetic beads. After washing, RNAs bound to hnRNPA1 were eluted and quantified. RT-PCR and real-time PCR were performed to examine whether miR-223-3p was co-immunoprecipitated with the hnRNPA1 antibody and results were expressed as fold enrichment of miR-223-3p.

### Reporter assay

Cells were seeded in six-well plates and allowed for 24 hr attachment. Followed by which co-transfection was performed with (miR-223-3p mimics and LMO2 3′UTR reporter vector) or (miR-138-5p mimics and BCL11A 3′UTR reporter vector) using Lipofectamine 2000 (Invitrogen); 1 μg of LMO2 or BCL11A 3′UTR or control vector and 100 nM miR-223 or miR-138-5p mimics or scramble mimics was added per well. After 24 hr, cell lysates were measured for luciferase activity using Dual-Luciferase Reporter Assay System (Promega).

### Statistical analysis

The mean and standard deviation were calculated for each of the determined parameters. Error bars represent standard deviation (SD) of a triplicate set of experiments. Statistical analyses were performed using unpaired Student’s t-test. The level of statistical significance was set at *P < 0.05, **P < 0.01, ***P < 0.001, ****P < 0.0001.

**Note:** Please see Supplementary Data S1 for details regarding primers and reporter constructs used; Supplementary Data S2 for uncropped western blots. Supplementary Data S3 shows the heatmap of differentially expressed exo-miRNAs from β-TM and HD.

## Electronic supplementary material

Supplementary Information
