# Supplementary material for: Reciprocal regulation of γ-globin expression by exo-miRNAs: Relevance to γ-globin silencing in β-thalassemia major
Source: Sci Rep. 2017 Mar 16;7:202. doi: 10.1038/s41598-017-00150-7 (PMC5427890; doi:10.1038/s41598-017-00150-7)
Supplement: Supplementary file 1 — Supplementary Information [file 41598_2017_150_MOESM1_ESM.pdf]

Reciprocal regulation of γ-globin expression by exo-miRNAs: Relevance to γ-globin silencing in β-thalassemia major

Kuo-Ting Sun MD PhD[1,2,3], Yu-Nan Huang MSc[1,4,7#], Kalai Selvi Palanisamy PhD[1#], Shih-Sheng Chang MD[1,5], I-Kuan Wang MD PhD[1,6], Kang-Hsi Wu MD[7], Ping Chen MD PhD[8], Ching-Tien Peng MD[7]*, Chi-Yuan Li MD[1,9]*

Supplementary Data S1: Lists of primers and constructs used for over expression, sponge and mutation analysis

miRNA qRT-PCR primers

miRNA R-primer
GTGCAGGGTCCGAGGT

hsa-miR-223-3p-F
CCCTGTCAGTTTGTCAAAT

hsa-miR-223-3p-RT
GTTGGCTCTGGTGCAGGGTCCGAGGTATTCGCACCAGAGCCAACTGGGGT

hsa-snoRNA95-F
GTGCTGAAATCCAGAGGCT

hsa-snoRNA95-RT
GTTGGCTCTGGTGCAGGGTCCGAGGTATTCGCACCAGAGCCAACGCTCAG

To clone LMO2 3'UTR or to make mutation constructs

hsa-LMO2 3'UTR-WT-F (PmeI)
AAGTTTAAACGCCCGAGTCCCCGGGCATCT

hsa-LMO2 3'UTR-WT-R (XhoI)
GTCCCTCGAGTTTCAGTCTGTCATTTTTAT

hsa-LMO2 3'UTR-MT -F
CCATCCATAGTTTGACTGTTGATTAGCAGA

hsa-LMO2 3'UTR-MT-R
TCTGCTAATCAACAGTCAAACTATGGATGG

To clone BCL11A 3'UTR or to make mutation constructs

hsa-BCL11A 3'UTR-WT-F (PmeI)
AAGTTTAAACAGGTATATTAATACCCCTCC

hsa-BCL11A 3'UTR-WT-R (XhoI)
GTCCCTCGAGCGAATTAGGGACAATTTAAA

hsa-BCL11A 3'UTR-MT -F
AGAGTGCTTGTGTGGTCGTCACCTGTTTTT

hsa-BCL11A 3'UTR-MT-R
AAAAACAGGTGACGACCACACAAGCACTCT

To clone viral miR-223 overexpression constructs

hsa-miR-223stemloop-F (PmeI)
AAGTTTAAACGGGCTTTACCTGCTTATCTT

hsa-miR-223stemloop-R (EcoRI)
AAGAATTCTGTGTAGACACAGCCCAGGGCT

To clone viral miR-223 sponge constructs

hsa-miR-223-spongeX4-F (PmeI)
AAGTTTAAACACCTCTGTACAGAGAATACA

hsa-miR-223-spongeX4-R (EcoRI)
AAGAATTCTATACACAGCTTATAGAACT

To clone stem-loop of pri-miR-223 reporter or to make mutation constructs

hsa-miR-223stemloop-F (PmeI)
AAGTTTAAACGGGCTTTACCTGCTTATCTT

hsa-miR-223stemloop-R (XhoI)
GTCCCTCGAGTGTGTAGACACAGCCCAGGGCT

hsa-miR-223stemloop-MT-F
CTCCATGACCATCTGTGTCAGT

hsa-miR-223stemloop-MT-R
ACTGACACAGATGGTCATGGAG

Supplementary Table S1: Motifs of HnRNPs family

RBPs	Motifs
HNRNPA1	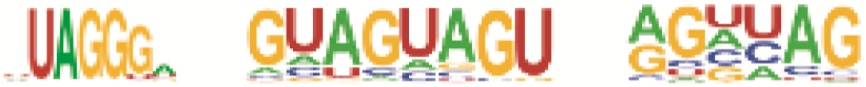
HNRNPA1L2	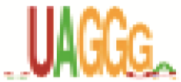
HNRNPA2B1	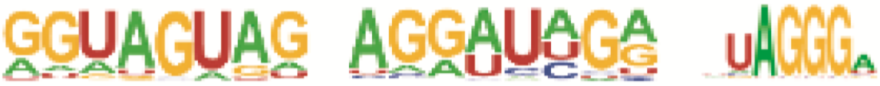
HNRNPC	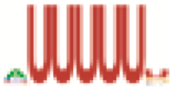
HNRNPCL1	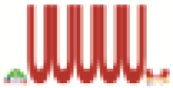
HNRNPF	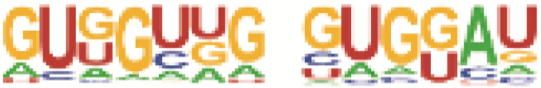 GGGUG
HNRNPH1	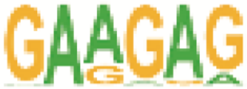
HNRNPH2	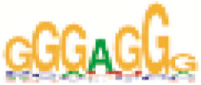
HNRNPK	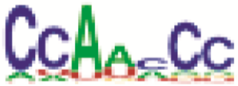
HNRNPL	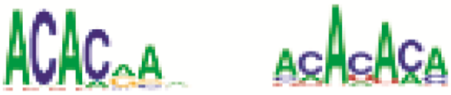
HNRNPLL	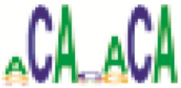
HNRNPM	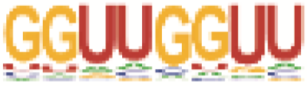
HNRNPU	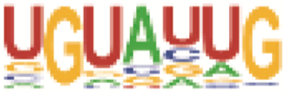

Supplementary Data S2: Uncropped western blots

1c

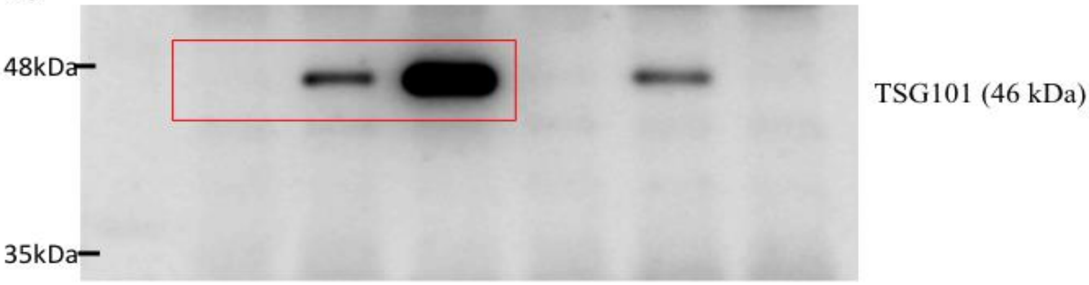

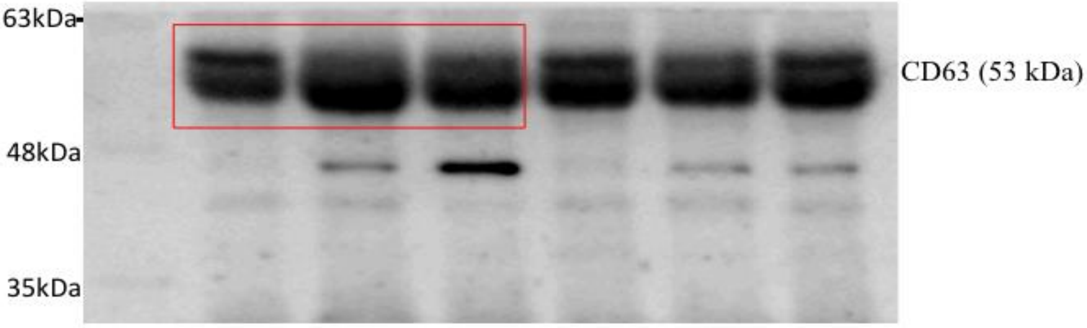

3d

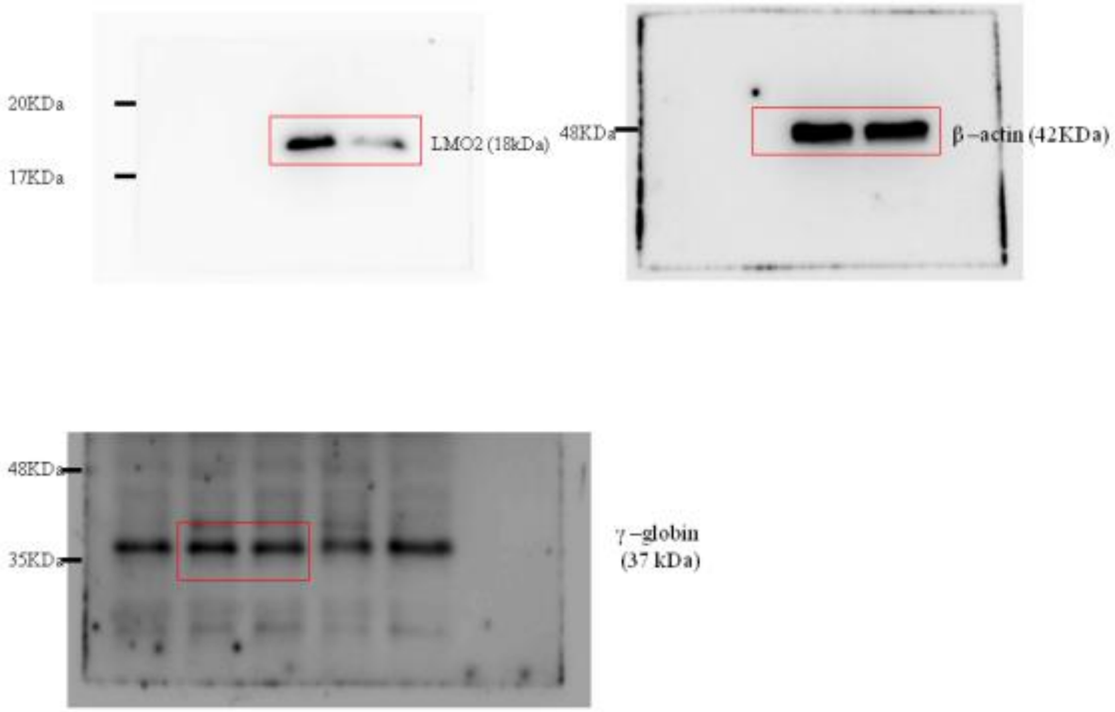
20KDa
17KDa
LMO2 (18kDa)
48KDa
β–actin (42KDa)
48KDa
35KDa
γ–globin
(37 kDa)

4d

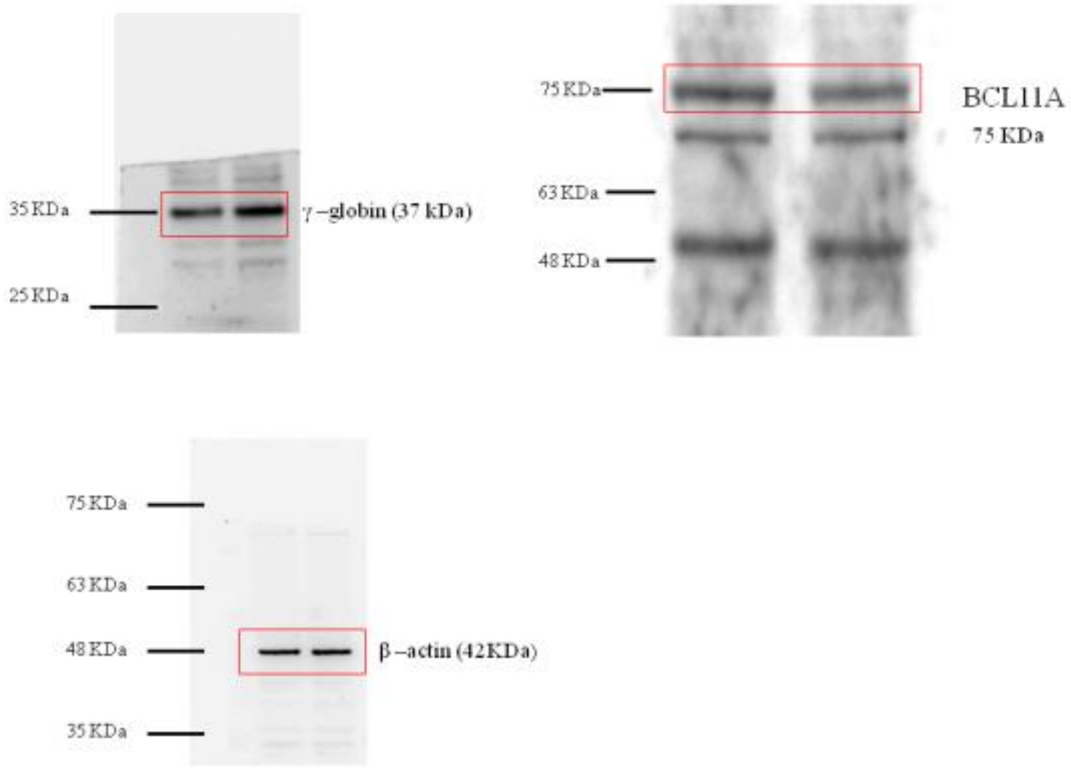
35KDa
25KDa
γ–globin (37 kDa)
75KDa
63KDa
48KDa
BCL11A
75 KDa
75KDa
63KDa
48KDa
35KDa
β–actin (42KDa)

5b

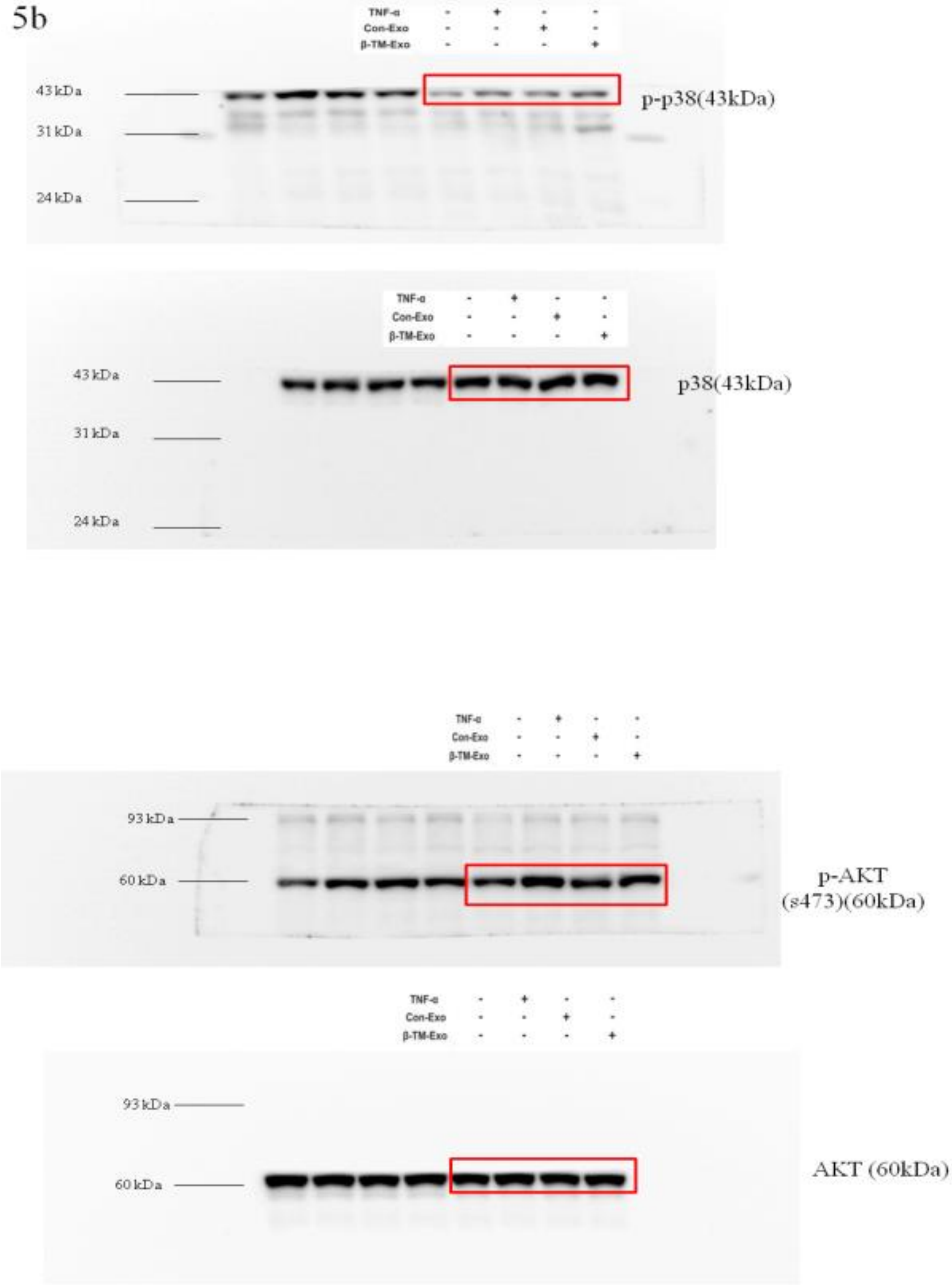
TNF-α - + - -
Con-Exo - - + -
β-TM-Exo - - - +
43 kDa
31 kDa
24 kDa
p-p38(43kDa)
TNF-α - + - -
Con-Exo - - + -
β-TM-Exo - - - +
43 kDa
31 kDa
24 kDa
p38(43kDa)
TNF-α - + - -
Con-Exo - - + -
β-TM-Exo - - - +
93 kDa
60 kDa
p-AKT (s473)(60kDa)
TNF-α - + - -
Con-Exo - - + -
β-TM-Exo - - - +
93 kDa
60 kDa
AKT (60kDa)

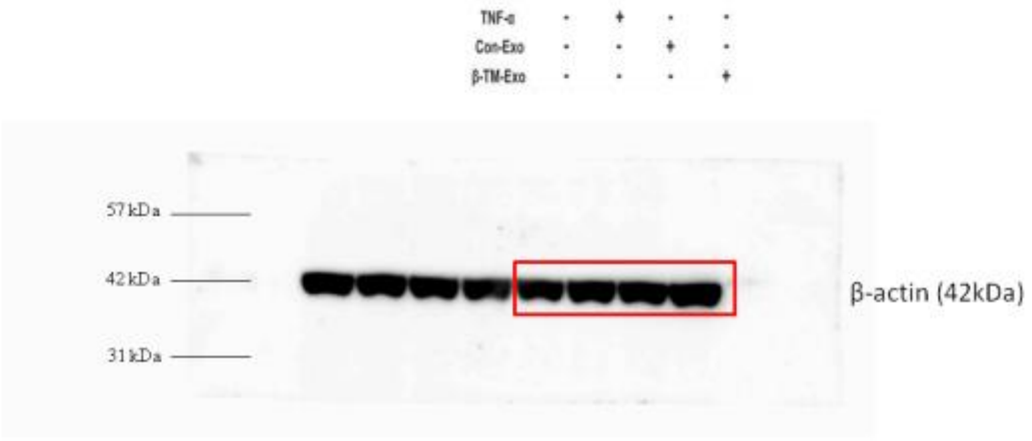
TNF-α
Con-Exo
β-TM-Exo
57 kDa
42 kDa
31 kDa
β-actin (42kDa)

5c

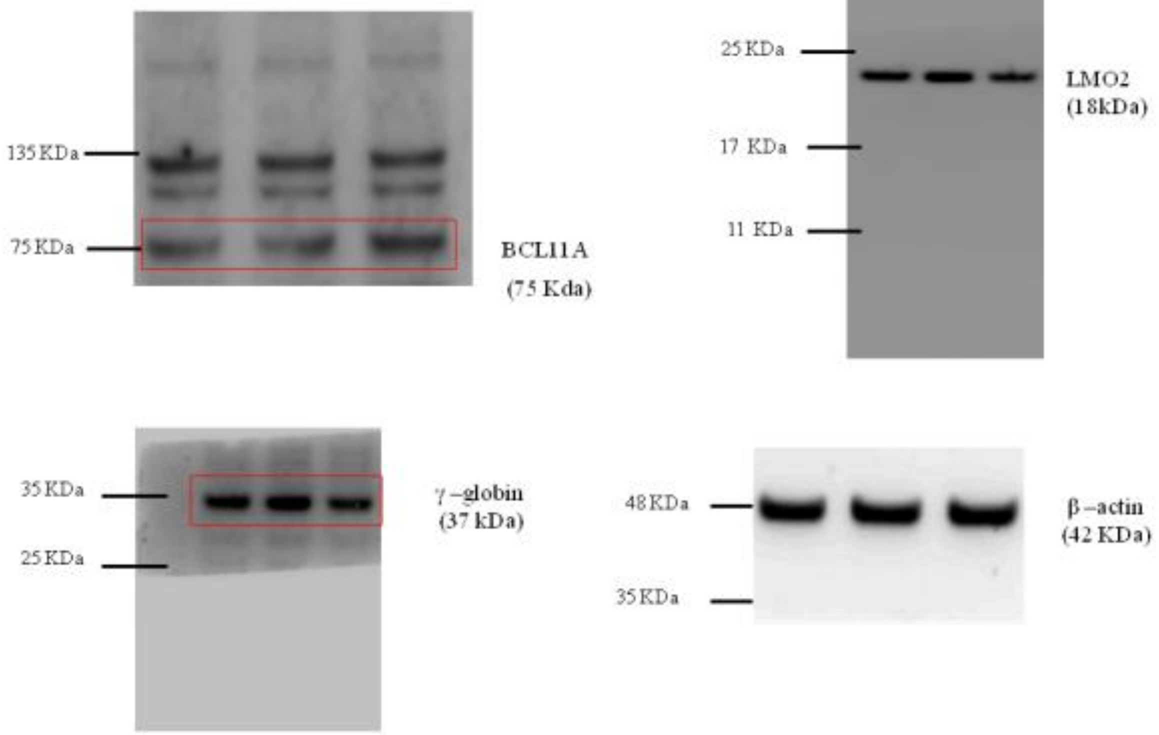
135 KDa
75 KDa
BCL11A
(75 Kda)
25 KDa
17 KDa
11 KDa
LMO2
(18kDa)
35 KDa
25 KDa
γ -globin
(37 kDa)
48 KDa
35 KDa
β -actin
(42 KDa)

6c

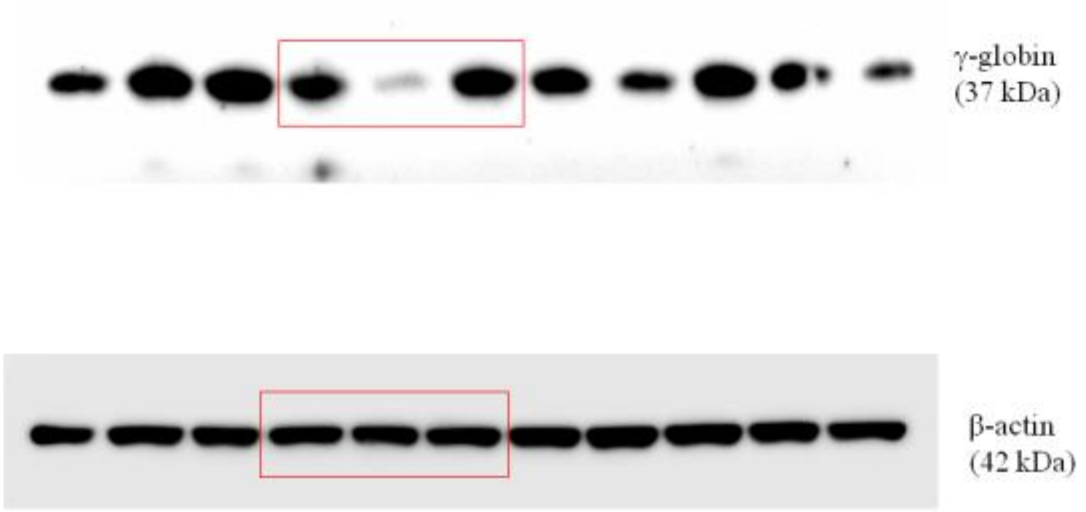

7c

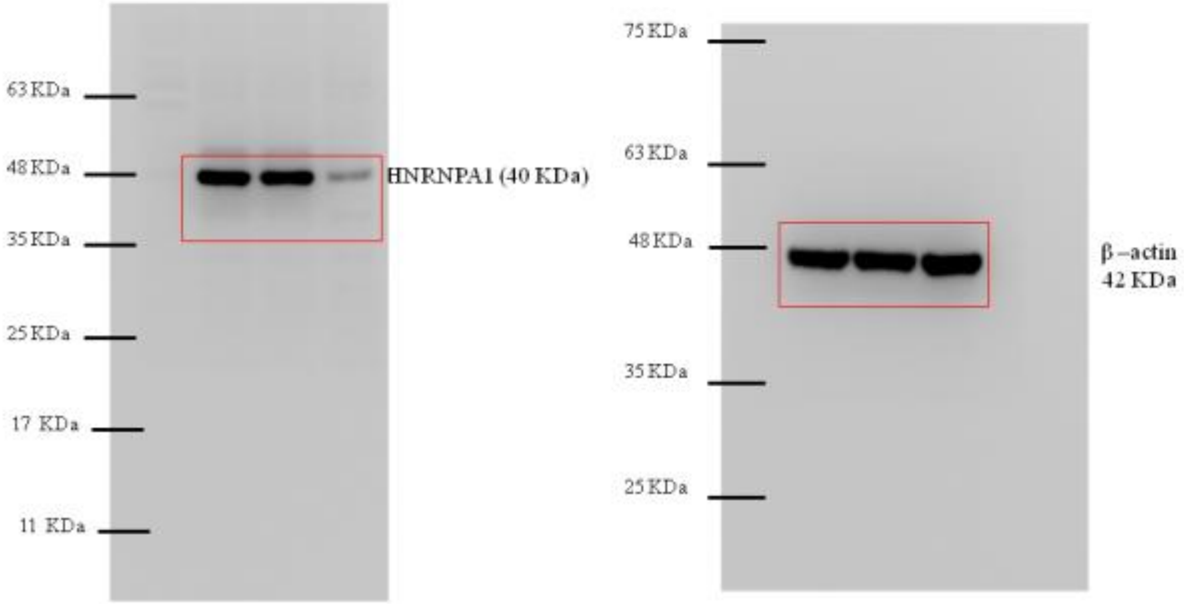

Supplementary Data S3: Heatmap of differentially expressed exo-miRNAs

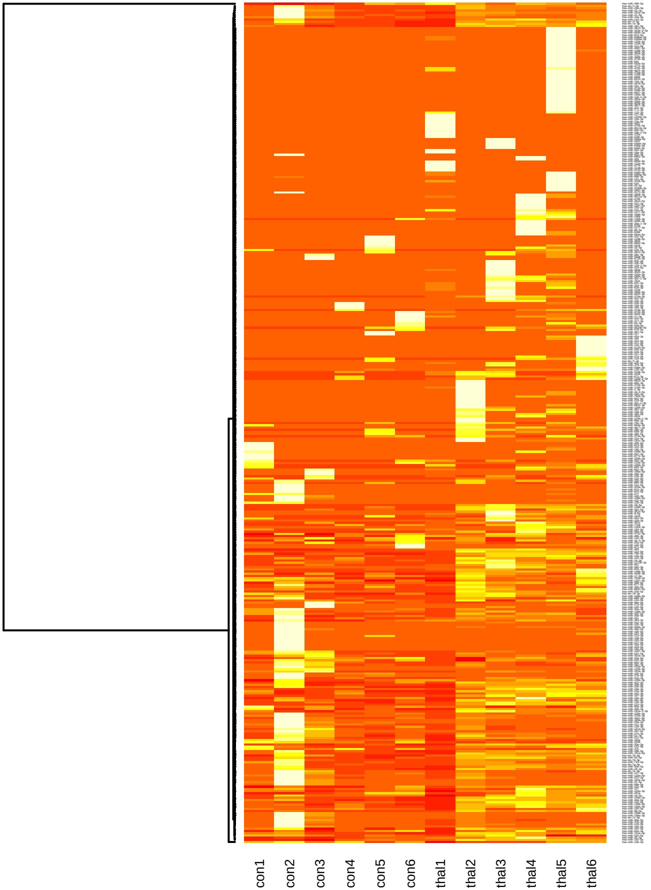
con1
con2
con3
con4
con5
con6
thal1
thal2
thal3
thal4
thal5
thal6
